# Effects of Low-Protein Diets on Growth Performance, Intestinal Morphology, Apparent Ileal Amino Acid Digestibility and Jejunal Amino Acid Transporter Gene Expression in Heat-Stressed Growing Male Pekin Ducks †

**DOI:** 10.3390/ani16081247

**Published:** 2026-04-18

**Authors:** Zhongjian Shen, Han Bao, Haoran Zhang, Dan Yuan, Wei Huang, Shuisheng Hou, Ming Xie, Meizhi Wang

**Affiliations:** 1 College of Animal Science and Technology, China Agricultural University, Beijing 100193, China; zhong96jian@163.com; 2 State Key Laboratory of Animal Science, Institute of Animal Sciences, Chinese Academy of Agricultural Sciences, Beijing 100193, China; 13574815197@163.com (H.B.); zhanghaoran0506@163.com (H.Z.); yuandan126@163.com (D.Y.); hw-1115@163.com (W.H.); houss@263.net (S.H.)

**Keywords:** duck, heat stress, low-protein diet, growth performance, amino acid transporter

## Abstract

The present study investigated the effects of low-protein (LP) diets on growth performance, intestinal morphology, apparent ileal digestibility (AID), and jejunal amino acid (AA) transporter mRNA expression in heat-stressed Pekin ducks. A total of 108 fourteen-day-old male ducks were randomly allotted to three treatments, each with six replicates of six birds: a thermoneutral (TN) group fed a control (CON) diet, a heat stress (HS) group fed the CON diet, and an HS-LP group fed the LP diet. Ducks were maintained at 22 °C (TN) or 30 °C (HS and HS-LP) for 3 wk. The CON and LP diets contained 17.5% and 16.0% CP, respectively, with similar AA profiles. Compared with the TN group, the HS group showed reduced BW, ADFI, and ADG and increased F:G (*p* < 0.05). Compared with the HS group, the HS-LP group showed higher BW and ADG and lower F:G (*p* < 0.05), although BW and ADG remained lower than in the TN group. HS decreased villus height (VH), jejunal villus height-to-crypt depth (VH:CD) ratios, and goblet cell counts (*p* < 0.05). Compared with the HS group, the HS-LP group showed higher jejunal and ileal VH:CD ratios and lower ileal CD (*p* < 0.05), whereas VH and goblet cell counts were not restored to TN values. Although AID of CP and AAs did not differ between the TN and HS groups, the HS-LP group showed higher AID of CP and AAs than the HS group (*p* < 0.05). In addition, the HS-LP group showed lower *PEPT1* and higher *LAT1* mRNA expression (*p* < 0.05). In conclusion, a reduction in dietary CP from 17.5% to 16.0% partially alleviated HS-induced growth depression and intestinal morphological impairment and was accompanied by increased AID of AAs and altered mRNA expression of jejunal AA transporters.

## Simple Summary

High environmental temperatures can impair growth performance and intestinal morphology in meat ducks, posing a challenge to poultry production. Lowering dietary protein while supplying essential amino acids has been proposed as a potential nutritional strategy, but its effectiveness under heat stress remains unclear. This study examined how a moderate reduction in dietary protein affects growth performance, intestinal morphology, and nutrient utilization in ducks exposed to high temperatures. The results showed that heat stress reduced growth performance and feed intake, whereas reducing dietary protein from 17.5% to 16.0% while maintaining a balanced amino acid supply partially improved growth performance and intestinal morphology, but these responses were not fully restored to thermoneutral levels. This approach also increased apparent ileal amino acid digestibility and altered the mRNA expression of genes involved in intestinal amino acid absorption. Overall, moderate protein reduction partially mitigated some adverse effects of heat stress in ducks raised under hot conditions, despite not fully restoring all responses to thermoneutral levels.

## 1. Introduction

Global warming is increasingly posing significant challenges to modern poultry production [1,2]. Pekin ducks, a fast-growing meat poultry species, are particularly susceptible to high temperatures due to their thick feather coverage and additional down layer. When ambient temperatures exceed their thermal comfort zone, ducks experience heat stress (HS), which suppresses growth and increases mortality [3,4]. Extensive research has shown that HS impairs poultry performance by reducing feed intake and damaging intestinal morphology [5,6,7,8]. The intestine relies on mucin secreted by goblet cells to maintain mucosal barrier integrity [9]. HS further compromises this defense by reducing intestinal oxygen and nutrient supply [10,11], leading to structural damage such as villus atrophy and mucosal injury [12,13,14,15].

Low-protein (LP) diets have been proposed as a nutritional strategy to alleviate the negative effects of HS. By partially replacing intact protein with crystalline AAs, LP diets increase the proportion of non-protein-bound AAs that can be rapidly absorbed in the intestine [16,17]. Because the jejunum is a major site of intestinal AA and peptide absorption, changes in jejunal transporter expression may be relevant to dietary AA utilization [18]. AA absorption is mediated by specific transporters expressed in enterocytes, including PEPT1 (encoded by *SLC15A1*), which primarily transports di- and tripeptides; LAT1 (*SLC7A5*) and LAT4 (*SLC43A2*), which are involved in the transport of large neutral AAs; B^0^AT1 (*SLC6A19*), which mediates neutral AA transport; CAT1 (*SLC7A1*), which mediates cationic AA transport; and b^0,+^AT (*SLC7A9*), which is involved in neutral and cationic AA transport [19]. Previous studies in poultry indicate that intestinal nutrient transporter expression can be influenced by intestinal segment, dietary substrate availability, and stress conditions [20,21]. Therefore, heat stress and dietary protein supply may affect the expression of these jejunal AA transporters. In the present study, the mRNA abundance of *SLC15A1*, *SLC7A5*, *SLC7A9*, *SLC7A1*, *SLC43A2*, and *SLC6A19* was measured to provide transcriptional evidence related to intestinal AA absorption under HS and LP feeding conditions. In addition, dietary protein contributes more to metabolic heat production than fats or carbohydrates due to its higher heat increment; therefore, reducing dietary protein intake can decrease diet-induced thermogenesis and improve thermal tolerance under HS [22,23]. Excessive protein restriction, however, may impair growth performance even though it can reduce mortality [23,24,25]. Recent studies have shown that a moderate reduction in dietary crude protein (CP) from 16.0% to 14.5%, coupled with adequate supplementation of Lys, Met, and Thr, improves both intestinal morphology and growth performance in heat-stressed yellow-feathered broilers [26]. Similar improvements have also been reported in quails fed AA-balanced LP diets under HS conditions [27]. However, evidence regarding the effectiveness of LP diets in alleviating HS in Pekin ducks remains limited, and in particular, little is known about how elevated ambient temperature and dietary protein reduction affect intestinal AA digestibility and transporter transcriptional responses in ducks. Previous studies conducted under thermoneutral (TN) conditions indicate that dietary CP can be reduced from 17.5% to 16.0% when crystalline AAs, including Lys, Met, Thr, Trp, Arg, Ile, Val, and Gly, are supplemented to meet standardized ileal digestible (SID) AA requirements without compromising growth performance in growing Pekin ducks [28,29]. Whether reducing dietary CP from 17.5% to 16.0% can alleviate the HS response in Pekin ducks remains unclear.

Therefore, the objectives of the present study were to evaluate the effects of elevated ambient temperature on growth performance, intestinal morphology, apparent ileal digestibility (AID) of AAs and CP, and jejunal AA transporter gene expression in growing Pekin ducks and to determine whether reducing dietary CP from 17.5% to 16.0% could mitigate the adverse effects of HS.

## 2. Materials and Methods

### 2.1. Experimental Design and Procedure

In our study, a total of 150 one-day-old male Pekin ducks were housed from hatch to 14 days of age in wire-floor pens (1.4 m × 0.6 m; 15 ducks per pen) and fed a common starter diet containing 2900 kcal/kg metabolizable energy and 20% CP. During this period, the ambient temperature was maintained at 32 °C for the first three days and gradually decreased to 26 °C by day 14.

At 14 days of age, 108 ducks were randomly selected and randomly allocated to three treatment groups, with six pens per treatment and six ducks per pen (0.8 m × 0.8 m). The thermoneutral (TN) group was maintained at a constant ambient temperature of 22 ± 1 °C and fed a control (CON) diet. The HS group was continuously exposed to an ambient temperature of 30 ± 1 °C and fed the CON diet. The HS-LP group was continuously exposed to an ambient temperature of 30 ± 1 °C and fed an LP diet. The experimental period lasted for 3 wk, and relative humidity was maintained at 60% across all treatments. The composition and nutrient levels of the experimental diets are detailed in Table 1. The CON diet contained 17.5% CP, while the LP diet contained 16.0% CP. Both diets met the AA requirements for Pekin ducks according to NRC recommendations, with matched levels of SID Lys and a similar AA pattern, including supplementation with crystalline Lys, Met, Cys, Thr, Trp, Arg, Ile, Val, and Gly. All diets were pelleted (the starter diet as 2.5 mm diameter pellets and the experimental diets as 4.0 mm diameter pellets) and provided ad libitum, with water available via drip-nipple drinkers under continuous lighting (20 lux/m^2^).

### 2.2. Growth Performance and Sample Collection

At 32 days of age, body weight (BW), average daily feed intake (ADFI), average daily gain (ADG), and feed-to-gain ratio (F:G) were determined on a per-pen basis.

At 35 days of age, all ducks were euthanized by carbon dioxide asphyxiation. One duck with a body weight closest to the pen average was selected from each pen for intestinal morphology and jejunal mucosa sampling. Intestinal segments (1–2 cm in length) were collected from the middle portion of the jejunum (between the end of the duodenal loop and Meckel’s diverticulum) and from the first third of the ileum (between Meckel’s diverticulum and approximately 1 cm anterior to the ileocecal junction). These segments were rinsed gently with saline and fixed in 4% paraformaldehyde for histological analysis. An approximately 2 cm segment from the same middle portion of the jejunum was collected, gently rinsed with physiological saline, and the mucosa was carefully scraped, collected into 1.5 mL RNase-free cryo tubes, snap-frozen in liquid nitrogen, and stored at −80 °C for subsequent mRNA analysis of AA transporters. Furthermore, ileal digesta from the distal two-thirds of the ileum were collected from all ducks within each pen, pooled into a single 50 mL centrifuge tube per pen, flushed with distilled water, and stored at −20 °C for the determination of the AID of CP and AAs.

### 2.3. Intestinal Morphology

Fixed intestinal samples were dehydrated, embedded in paraffin, sectioned to 5 μm thickness, and stained with Alcian blue. Villus height (VH), crypt depth (CD), and the number of goblet cells per villus were measured in both the jejunum and ileum using a light microscope (Olympus Inc., Tokyo, Japan) equipped with a computer-assisted morphometric system and Image-Pro Plus 6.0 software (Media Cybernetics, Rockville, MD, USA). VH was defined as the distance from the villus tip to the villus–crypt junction, while CD was measured from the crypt base to the same point. All measurements were averaged from 20 villi per sample.

### 2.4. AID of CP and AAs

Ileal digesta samples were freeze-dried, finely ground, and then passed through a 40-mesh sieve. The dry matter and N contents of diets and digesta were determined according to the methods of AOAC [30]. The concentrations of 16 AAs (Lys, Met, Cys, Thr, Arg, Ile, Val, Gly, Asp, Ser, Glu, Pro, Ala, Leu, Phe, and His) were determined according to the method described by Xie et al. [28] and analyzed using an AA analyzer (Biochrom 30, Biochrom Ltd., Cambridge, UK). Acid-insoluble ash (AIA) was measured following the method of Liu [31] and used as an internal marker to calculate AID. The formula for AID is as follows:

AID (%) = [1 − (T_digest_ × M_diet_)/(T_diet_ × M_digest_)] × 100

T_diet_ = nutrient (AA or CP) concentration in the diet, T_digest_ = nutrient (AA or CP) in the digesta, M_diet_ = AIA concentration in the diet, M_digest_ = AIA concentration in the digesta.

### 2.5. Quantitative Real-Time PCR Analysis

Total RNA was extracted from frozen jejunal mucosa with RNAiso reagent (Code No. R401, Vazyme, Nanjing, China) according to the manufacturer’s instructions. The concentration and quality of the RNA were determined at 230 nm and 260/280 nm, respectively. Total RNA was reverse transcribed into cDNA using the PrimeScript™ RT Reagent Kit (Code No. RR092A, TaKaRa, Dalian, China) following the manufacturer’s instructions. Quantitative PCR was performed with the SYBR Premix Ex Taq™ II (Code No. CN830A, TaKaRa, Dalian, China) on a fluorescent detection system (Applied Biosystems QuantStudio 7 Flex, Thermo Fisher Scientific, Waltham, MA, USA). Target gene expression was normalized to the *β-actin* reference gene, and relative mRNA expression levels were calculated using the 2^−△△Ct^ method. Primer sequences utilized for qRT-PCR analysis are detailed in Table 2.

### 2.6. Statistical Analysis

The pen was considered the experimental unit for all analyses. For intestinal morphology and gene expression analyses, one duck per pen was randomly selected, whereas ileal digesta samples were pooled by pen. Data were analyzed using one-way ANOVA with the GLM procedure in SAS 9.4 (SAS Institute Inc., Cary, NC, USA), with treatment (TN, HS, and HS-LP) as the fixed factor. Prior to ANOVA, normality and homogeneity of variances were assessed using the Shapiro–Wilk and Levene’s tests, respectively. When a significant treatment effect was detected (*p* < 0.05), differences among means were evaluated using Duncan’s multiple range test.

## 3. Results

### 3.1. Growth Performance

As shown in Table 3, treatment significantly affected BW, ADFI, ADG, and F:G (*p* < 0.05). Compared with the TN group, the HS and the HS-LP groups exhibited lower BW, ADFI, and ADG (*p* < 0.05). Compared with the HS group, the LP diet significantly increased BW and ADG (*p* < 0.05), while no significant difference was observed in ADFI (*p* > 0.05). In addition, ducks in the HS group had a higher F:G ratio than those in the TN and HS-LP groups (*p* < 0.05), with no significant difference between the latter two groups (*p* > 0.05).

### 3.2. Intestinal Morphology

As shown in Table 4, treatment significantly affected jejunal VH, VH:CD ratio, and goblet cell counts, as well as ileal VH, CD, VH:CD ratio, and goblet cell counts (*p* < 0.05), but did not significantly affect jejunal CD (*p* > 0.05).

In the jejunum, VH and goblet cell counts were significantly lower in both the HS and HS-LP groups than in the TN group (*p* < 0.05), with no significant difference between the HS and HS-LP groups (*p* > 0.05). Consequently, the VH:CD ratio was significantly reduced in the HS group compared with both the TN and HS-LP groups (*p* < 0.05), with no significant difference between the latter two groups (*p* > 0.05).

In the ileum, VH and goblet cell counts were also significantly decreased in the HS and HS-LP groups compared with the TN group (*p* < 0.05), with no significant difference between the HS and HS-LP groups (*p* > 0.05). Ileal CD was significantly reduced in the HS-LP group compared with both the TN and HS groups (*p* < 0.05), with no significant difference between the latter two groups (*p* > 0.05). As a result, the VH:CD ratio was significantly lower in the HS group than in the HS-LP group (*p* < 0.05), while the TN group did not differ significantly from either the HS or HS-LP group (*p* > 0.05).

### 3.3. AID of CP and AAs

The AID of CP and AAs (Lys, Met, Cys, Thr, Arg, Ile, Val, Gly, Asp, Ser, Glu, Pro, Ala, Leu, Phe, and His) is presented in Table 5. Treatment significantly affected the AID of CP and all measured AAs (*p* < 0.05). No significant differences were observed between the TN and HS groups in the AID of CP or any measured AAs (*p* > 0.05). In contrast, the HS-LP group showed significantly higher AID of CP and all measured AAs than both the TN and HS groups (*p* < 0.05).

### 3.4. mRNA Expression of AA Transporters

**Figure 1:**
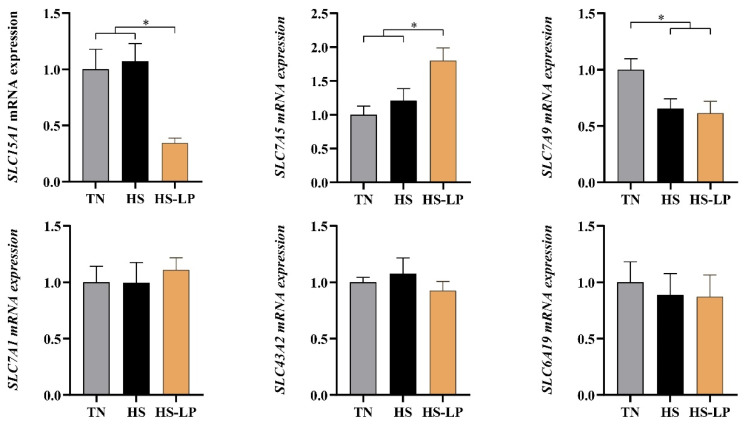
Effects of low-protein diets on the mRNA expression of AA transporters in the jejunum of 35-day-old ducks under heat stress. The analyzed genes include *SLC15A1* (*PEPT1*), peptide transporter; *SLC7A5* (*LAT1*), large neutral AA transporter; *SLC7A9* (*b*^0,+^*AT*), Na^+^-independent cationic and zwitterionic AA transporter; *SLC7A1* (*CAT1*), Na^+^-independent cationic AA transporter; *SLC43A2* (*LAT4*), Na^+^-independent basolateral neutral AA transporter; and *SLC6A19* (*B*^0^*AT1*), Na^+^-dependent neutral AA transporter. TN, thermoneutral group (22 ± 1 °C) + control diet (CP, 17.5%); HS, heat stress group (30 ± 1 °C) + control diet (CP, 17.5%); HS-LP, heat stress (30 ± 1 °C) + LP diet group, low-protein diet (CP, 16.0% + SID AA profile similar to the control diet). All values are expressed as mean ± SEM, *n* = 6 per treatment. Asterisks on the data bars indicate when *p* < 0.05 (*).

The relative mRNA expression of jejunal AA transporters is presented in Figure 1. Treatment significantly affected the expression of *SLC15A1* (*PEPT1*), *SLC7A5* (*LAT1*), and *SLC7A9* (*b*^0,+^*AT*) (*p* < 0.05), but did not significantly affect the expression of *SLC7A1* (*CAT1*), *SLC43A2* (*LAT4*), and *SLC6A19* (*B*^0^*AT1*) (*p* > 0.05). *PEPT1* mRNA expression was significantly lower in the HS-LP group than in the TN and HS groups (*p* < 0.05), with no significant difference between the latter two groups (*p* > 0.05). In contrast, *LAT1* mRNA expression was significantly higher in the HS-LP group than in the TN and HS groups (*p* < 0.05), with no significant difference between the latter two groups (*p* > 0.05). *b*^0,+^*AT* mRNA expression was significantly reduced in both the HS and HS-LP groups compared with the TN group (*p* < 0.05), with no significant difference between the HS and HS-LP groups (*p* > 0.05).

## 4. Discussion

In our study, Pekin ducks exposed to high ambient temperature (30 °C) showed significantly lower BW at 32 days of age, reduced ADG and ADFI from 14 to 32 days of age, and a higher F:G ratio. These responses are consistent with previous studies suggesting that reduced feed intake is an important factor contributing to HS-induced growth depression in poultry [32,33]. Pair-feeding analyses have further shown that a substantial proportion of the growth loss under HS may be attributable to reduced nutrient intake, although HS-related metabolic and physiological disturbances may also contribute [16,34]. Therefore, the impaired performance observed in the present HS group may have been associated mainly with the lower daily intake of energy and N, together with the additional maintenance costs imposed by HS [3,35,36].

Notably, HS in the present study was associated with impaired intestinal morphology, as evidenced by decreased VH, VH:CD ratio, and goblet cell counts. These morphological alterations are consistent with previous reports indicating that HS not only impairs growth performance but also induces intestinal damage [10,14,37,38,39]. However, no significant differences were observed between the TN and HS groups in the AID of CP or AAs. These variables reflect different aspects of intestinal function, with morphological indices indicating local mucosal structure and AID reflecting the net outcome of nutrient utilization at the distal ileum. In this context, reviews and comparative HS studies in poultry indicate that changes in nutrient digestibility are often limited and inconsistent, with the magnitude of the response depending in part on the severity and pattern of heat exposure and that these changes may account for only a relatively small proportion of the reduction in feed efficiency under HS [16,40]. Therefore, morphological alterations do not necessarily translate into proportional changes in AID, particularly when reduced nutrient intake lowers the luminal nutrient load, and the remaining absorptive capacity is still sufficient to maintain overall AID. Consistent with our findings, Habashy et al. [41] reported that continuous exposure to 35 °C did not affect the AID of CP in broilers, and Ghareeb et al. [42] observed no significant change in AA digestibility in heat-stressed broilers despite reduced feed intake. By contrast, Teyssier et al. [43] reported that constant HS reduced CP digestibility, whereas cyclic HS had no effect. Taken together, these findings suggest that HS-induced morphological impairment was not sufficient to cause a measurable reduction in AID under the present conditions. Moreover, the mRNA expression of most jejunal AA transporters remained unchanged under HS, suggesting that the transcriptional capacity for bulk AA uptake was largely preserved. Therefore, the reduced SLC7A9 expression may reflect a transporter-specific transcriptional response to HS rather than direct evidence of globally impaired AA absorption [19,41].

Despite its inability to increase feed intake or fully restore growth to the TN level under HS, the LP diet nevertheless partially alleviated growth depression. This response was observed when dietary CP was reduced from 17.5% to 16.0% while supplementing crystalline Lys, Met, Cys, Thr, Trp, Arg, Ile, Val, and Gly to meet AA requirements. The improved growth performance was accompanied by partial improvements in intestinal morphology, as reflected by increased jejunal and ileal VH:CD ratios, whereas goblet cell counts and villus height were unchanged. These findings indicate that, under HS conditions, the LP diet improved feed efficiency and some intestinal morphological indices but did not restore all physiological responses to the thermoneutral state. This partial benefit may be related, at least in part, to the lower heat increment of protein nutrition and the reduced metabolic burden associated with protein and AA catabolism under HS conditions [22,23]. Similar benefits of LP diets enriched with Lys, Met, and Thr have been reported in broilers under HS [26]. Of particular relevance, Cys, Thr, and Gly play critical roles in mucin synthesis, and their deficiency can lead to decreased goblet cell counts and reduced mucin secretion [44,45,46]. Thr and Gly supplementation has been shown to increase goblet cell counts in broilers, particularly Gly, which has been reported to exert beneficial effects on the intestinal mucosa under HS [47,48,49,50]. However, because goblet cell counts were not improved by the LP diet in the present study, the contribution of these supplemented AAs to mucosal protection under our experimental conditions remains uncertain. Future research should investigate whether increasing AA concentrations in LP diets could further enhance goblet cell proliferation and intestinal health in heat-stressed ducks. Moreover, because a thermoneutral LP group was not included in the present study, it remains unclear whether these responses were specific to HS conditions or reflected a general effect of the LP diet. Sex-related differences in intestinal responses to chronic HS have also been reported in Pekin ducks [15]. Accordingly, caution is warranted when extrapolating the present findings to female ducks.

Furthermore, the LP diet significantly increased the AID of CP and all measured AAs in heat-stressed ducks. This response was likely related, at least in part, to the greater proportion of crystalline AAs in the LP diet, which do not require prior hydrolysis and are generally absorbed more rapidly than protein-bound AAs in the small intestine [16,17]. Similar increases in AID with AA-balanced LP diets have also been reported in Pekin ducks and broilers under TN conditions [29,51,52,53,54], suggesting that the present response may partly reflect a broader effect of LP diet formulation rather than an effect unique to HS conditions. Therefore, the higher AID observed in HS-LP ducks is more appropriately interpreted as reflecting higher AID of dietary CP and AAs when a larger proportion is supplied in crystalline form rather than as direct evidence of a uniform enhancement in the overall digestive capacity of the intestine. Notably, the digestibility response to LP diets appears to vary with the extent of CP reduction and the formulation strategy used [53,55]. This point should be considered when interpreting the present findings because only one level of CP reduction was evaluated in the current study. The LP diet used here (16.0% CP) was selected because previous studies conducted under TN conditions showed that dietary CP could be reduced from 17.5% to 16.0% in growing Pekin ducks without compromising growth performance [28,29]. Therefore, the present results support the view that this level of CP reduction can partially improve growth performance and AID under HS, but they do not allow conclusions about whether a lower CP level would produce greater benefits or, conversely, impair performance under heat-stressed conditions. Further studies using multiple levels of dietary CP reduction are needed to clarify the dose–response relationship between the degree of CP reduction in LP diets, growth performance, and AID in heat-stressed meat ducks.

In parallel, the LP diet selectively altered the mRNA expression of jejunal AA transporters in heat-stressed ducks. In the present study, the mRNA expression of *SLC15A1* (*PEPT1*) was significantly reduced, whereas that of *SLC7A5* (*LAT1*) was significantly increased in the HS-LP group compared with the HS group, while the mRNA expression of most other transporters remained unchanged. These changes are more consistent with an adaptive, substrate-driven regulation. Similar responses to reduced dietary protein have been reported in broilers, in which LP diets altered the intestinal expression of nutrient transporters, including increased *y^+^LAT1* mRNA expression and reduced *PEPT1* mRNA expression in specific intestinal segments, suggesting compensatory adjustments in AA transport under protein-restricted conditions [56]. *PEPT1* primarily mediates di- and tripeptide transport, and its expression is influenced by dietary protein form and luminal peptide availability [57,58,59,60]. Because the LP diet partially replaced intact protein with crystalline AAs, fewer small peptides may have reached the jejunum, providing a plausible explanation for the lower *PEPT1* mRNA abundance. In contrast, *LAT1* is involved in the transcellular handling of large neutral AAs and may be upregulated when the intestinal AA supply pattern is altered [56]. Thus, the increased *LAT1* expression in HS-LP ducks may indicate an adaptive adjustment to the greater reliance on free large neutral AAs. Nevertheless, because only mRNA abundance was measured, these findings should be interpreted as transcriptional associations rather than direct evidence of altered transporter protein abundance or transport activity.

## 5. Conclusions

HS suppressed growth performance and impaired intestinal morphology in growing male Pekin ducks. Reducing dietary CP from 17.5% to 16.0% with crystalline AA supplementation partially alleviated HS-induced growth depression and intestinal morphological impairment but did not fully restore these responses to the TN level and was accompanied by increased AID of AAs and altered mRNA expression of selected jejunal AA transporters, including decreased *PEPT1* expression and increased *LAT1* expression.

## Figures and Tables

**Table 1:** Composition and nutrient levels of the corn–soybean meal experimental diets (%, as-fed basis).

Item	Diets ^1^	
	CON	LP
Ingredient, %		
Corn	68.0	71.8
Soybean meal	26.9	20.7
Soybean oil	0.8	1.1
Corn bran	-	1.2
Dicalcium phosphate	1.59	1.67
Limestone	1.22	1.24
Sodium chloride	0.30	0.30
L-Lysine HCl	0.05	0.21
DL-Methionine	0.14	0.16
L-Cystine	-	0.03
L-Threonine	-	0.08
L-Tryptophan	-	0.03
L-Arginine	-	0.19
Isoleucine	-	0.10
Valine	-	0.10
Glycine	-	0.09
Premix ^2^	1.00	1.00
Calculated nutrient levels ^3^, %		
AME (kcal/kg)	2900	2900
Crude protein	17.5 (17.54)	16.0 (16.16)
Calcium	0.86	0.86
Non-phytate phosphorus	0.40	0.40
Crude fiber	3.1	3.1
SID Lys	0.80 (0.89)	0.80 (0.86)
SID Met	0.39 (0.40)	0.39 (0.40)
SID Cys	0.30 (0.28)	0.30 (0.28)
SID Thr	0.61 (0.62)	0.61 (0.61)
SID Trp	0.17	0.17
SID Arg	1.12 (0.93)	1.12 (0.93)
SID Ile	0.66 (0.73)	0.66 (0.69)
SID Val	0.74 (0.83)	0.74 (0.79)
SID Gly	0.65 (0.70)	0.65 (0.68)

^1^ CON diet (control diet, 17.5% crude protein (CP)); LP diet (low-protein diet, 16.0% CP supplemented with crystalline amino acids (AAs) to match the standardized ileal digestible (SID) AA profile of CON diet). ^2^ Provided per kilogram of the final diet: Cu (CuSO_4_·5H_2_O), 10 mg; Fe (FeSO_4_·7H_2_O), 70 mg; Zn (ZnO), 80 mg; Mn (MnSO_4_·H_2_O), 80 mg; Se (NaSeO_3_), 0.3 mg; I (KI), 0.3 mg; choline chloride, 1000 mg; vitamin A, 9000 IU; vitamin D_3_, 2000 IU; vitamin E, 20 IU; vitamin K_3_, 2 mg; vitamin B_1_, 1 mg; vitamin B_2_, 6 mg; vitamin B_6_, 4 mg; vitamin B_12_, 0.01 mg; nicotinic acid, 50 mg; biotin, 0.2 mg; calcium-D-pantothenate, 20 mg; folic acid, 0.5 mg. ^3^ The values in parentheses are the analyzed CP and AA concentrations of the experimental diets.

**Table 2:** RT-PCR primer sequences.

Gene	Forward Sequences (5′-3′)	Reverse Sequences (5′-3′)
*β-actin*	GGTATCGGCAGCAGTCTTA	TTCACAGAGGGAGTAACTT
*SLC15A1*	TGACGCTCAGTTGCTGTTTG	CGCACCACCCATAGTGACAT
*SLC7A5*	TGGATCCCGAGAAGGACACT	GCAGGGTCATGATGCACGTA
*SLC7A9*	TGGCTCAGGTATCTTTGTTTCTCC	AACAAAGCGCCCCTAGTGT
*SLC7A1*	GGCTCTGCTACGGAGAGTTC	CACGCTGGAGGTTCCGATG
*SLC43A2*	CCACGAGTCGGGAAACTCC	GATGGTGAAGGCCAGGTTCA
*SLC6A19*	ATGTTGACGTGCGTAGGGTT	TCATGAAGGCTCCTCCTCCA

*β-actin* expression level was used as an internal control; *SLC15A1* (*PEPT1*), peptide transporter; *SLC7A5* (*LAT1*), large neutral AA transporter; *SLC7A9* (*b*^0,+^*AT*), Na^+^-independent cationic and zwitterionic AA transporter; *SLC7A1* (*CAT1*), Na^+^-independent cationic AA transporter; *SLC43A2* (*LAT4*), Na^+^-independent basolateral neutral AA transporter; and *SLC6A19* (*B*^0^*AT1*), Na^+^-dependent neutral AA transporter.

**Table 3:** Effects of low-protein diets on growth performance in heat-stressed ducks from 14 to 32 days of age ^1^.

Items	TN	HS	HS-LP	SEM	*p*-Value
BW (g) 14 d	594.5	593.0	594.5	0.87	0.745
BW (g) 32 d	2458.3 ^a^	2089.3 ^c^	2210.3 ^b^	42.68	<0.001
ADFI (g/d/bird)	222.0 ^a^	192.4 ^b^	196.7 ^b^	3.84	<0.001
ADG (g/d/bird)	103.6 ^a^	83.1 ^c^	89.8 ^b^	2.36	<0.001
F:G (g/g)	2.14 ^b^	2.31 ^a^	2.20 ^b^	0.024	0.002

^1^ Results are means with *n* = 6 per treatment. TN, thermoneutral group (22 ± 1 °C) + control diet (CP, 17.5%); HS, heat stress group (30 ± 1 °C) + control diet (CP, 17.5%); HS-LP, heat stress (30 ± 1 °C) + LP diet group, low-protein diet (CP, 16.0% + SID AA profile similar to the control diet). ^a–c^ Means with different superscripts within the same row differ significantly (*p* < 0.05).

**Table 4:** Effects of low-protein diets on intestinal morphology in 35-day-old ducks under heat stress ^1^.

Items	TN	HS	HS-LP	SEM	*p*-Value
Jejunum					
Villus height (μm)	842 ^a^	619 ^b^	725 ^b^	28.7	0.002
Crypt depth (μm)	217	212	180	7.5	0.093
VH:CD (μm/μm)	3.99 ^a^	3.01 ^b^	4.20 ^a^	0.178	0.008
Goblet cell (count/villus)	124.2 ^a^	98.3 ^b^	100.4 ^b^	3.23	<0.001
Ileum					
Villus height (μm)	713 ^a^	621 ^b^	634 ^b^	14.4	0.012
Crypt depth (μm)	168 ^a^	172 ^a^	132 ^b^	6.4	0.011
VH:CD (μm/μm)	4.33 ^ab^	3.72 ^b^	4.90 ^a^	0.181	0.021
Goblet cell (count/villus)	129.4 ^a^	106.0 ^b^	104.1 ^b^	3.76	0.004

^1^ Results are means with *n* = 6 per treatment. TN, thermoneutral group (22 ± 1 °C) + control diet (CP, 17.5%); HS, heat stress group (30 ± 1 °C) + control diet (CP, 17.5%); HS-LP, heat stress (30 ± 1 °C) + LP diet group, low-protein diet (CP, 16.0% + SID AA profile similar to the control diet). ^a,b^ Means with different superscripts within the same row differ significantly (*p* < 0.05).

**Table 5:** Effects of low-protein diets on apparent ileal digestibility of crude protein and AAs in 35-day-old ducks under heat stress ^1^.

Items, %	TN	HS	HS-LP	SEM	*p*-Value
CP	68.1 ^b^	65.3 ^b^	75.0 ^a^	1.37	0.002
Lys	69.2 ^b^	65.7 ^b^	77.5 ^a^	1.67	0.002
Met	84.7 ^b^	83.7 ^b^	88.6 ^a^	0.84	0.031
Cys	63.3 ^b^	60.5 ^b^	74.6 ^a^	1.75	<0.001
Thr	57.6 ^b^	53.9 ^b^	68.9 ^a^	1.96	<0.001
Arg	81.9 ^b^	79.8 ^b^	86.6 ^a^	1.01	0.008
Ile	73.6 ^b^	71.4 ^b^	80.9 ^a^	1.30	0.001
Val	71.1 ^b^	68.2 ^b^	78.4 ^a^	1.37	<0.001
Gly	59.1 ^b^	55.0 ^b^	71.2 ^a^	2.04	<0.001
Asp	70.0 ^b^	67.4 ^b^	76.5 ^a^	1.23	<0.001
Ser	70.9 ^b^	67.7 ^b^	76.3 ^a^	1.10	<0.001
Glu	78.2 ^b^	75.9 ^b^	82.5 ^a^	0.90	0.001
Pro	81.4 ^b^	79.8 ^b^	85.6 ^a^	0.79	<0.001
Ala	75.5 ^b^	73.0 ^b^	81.0 ^a^	1.14	0.003
Leu	78.0 ^b^	75.4 ^b^	82.6 ^a^	1.01	0.003
Phe	66.6 ^b^	62.2 ^b^	72.3 ^a^	1.39	0.003
His	74.3 ^b^	71.5 ^b^	79.2 ^a^	1.07	0.002

^1^ Results are means with *n* = 6 per treatment. TN, thermoneutral group (22 ± 1 °C) + control diet (CP, 17.5%); HS, heat stress group (30 ± 1 °C) + control diet (CP, 17.5%); HS-LP, heat stress (30 ± 1 °C) + LP diet group, low-protein diet (CP, 16.0% + SID AA profile similar to the control diet). Abbreviations: Lys, lysine; Met, methionine; Cys, cysteine; Thr, threonine; Arg, arginine; Ile, isoleucine; Val, valine; Gly, glycine; Asp, aspartic acid; Ser, serine; Glu, glutamic acid; Pro, proline; Ala, alanine; Leu, leucine; Phe, phenylalanine; His, histidine. ^a,b^ Means with different superscripts within the same row differ significantly (*p* < 0.05).

## Data Availability

The data in the current study are available from the corresponding authors.
